# Supplementary material for: Survival rate, blood feeding habits and sibling species composition of Aedes simpsoni complex: Implications for arbovirus transmission risk in East Africa
Source: PLoS Negl Trop Dis. 2022 Jan 24;16(1):e0010171. doi: 10.1371/journal.pntd.0010171 (PMC8812930; doi:10.1371/journal.pntd.0010171)
Supplement: Attachment — Submitted filename: Review.docx [file pntd.0010171.s001.docx]

Summary:

The goal of this paper is to evaluate the vectorial capacity of members of the Ae. simpsoni complex in two areas in Kenya: coastal Rabai and Rift Valley Kerio Valley), based on longevity and blood feeding habits. The authors analyze 539 mosquitos collected using CO2-baited BG-sentinel traps between 2019 and 2020, determine their longevity based on parity and use a fragment of the ITS2 to identify a subset of the samples (N=43) to subspecies. The authors also identify the blood-host of 24 blood-fed mosquitoes.

The data suggests high survival rates (longevity) for Ae. simpsoni in Kenya, and highlight that Ae. bromeliae is the dominant species in the region. This paper also reports the presence of Ae. lilii for first time in Rabai, Kenya. Blood-meal analysis indicates that members of this complex (mostly Ae. bromeliae) have a wide host feeding range, with moderate feeding from humans. The authors conclude based on longevity and moderate feeding on humans, that Ae. bromeliae has a high vectorial capacity for pathogen transmission. They also mention the possibility of having discovered a new cryptic species, but evidence for this is unclear.

Using basic molecular and morphological data, this manuscript provides important information on the ecology of the Ae. simpsoni complex in two sites of Kenya, which is relevant to evaluate vectorial capacity of these mosquitoes as vectors of disease. I find the results interesting and worth publishing; however, I have the concerns listed below.

Mayor comments:

1. Large numbers of mosquitoes were analyzed for parity and survival rate, yet mean longevity rate is reported without confidence intervals. It will be interesting to know the distribution of this parameter. Also, is it possible that the high survival rates derived from parity are consequence of a bias in the trapping method? Furthermore, the assumption of a 3-day gonotrophic cycle can significantly impact this metric, how realistic is this value? Can the authors estimate the length of the gonotrophic cycle under laboratory conditions? The citation provided in the text is for the gonotrophic cycle of a different mosquito species.

2. Out of the 539 mosquitoes screened for parity, a subset of 43 were used for species identification by ITS2 and used to construct the ML tree from figure 2. Then, another ML tree from ITS2 sequences is presented in Figure 4, this time including blood-fed specimens. It is not clear to me if the 24 blood-fed mosquitoes were included among the 43 individuals from Figure 2. I do not see a benefit on having two independent figures providing basically the same information. I suggest to combine all sequences from Figure 2 and 4 into one figure, with one ML tree and including the information of the host. Also, it is very difficult to see which samples belong to which clade from Figure 2, expanding the tree along the horizontal axis may help.

3. Unbalanced sampling. While subsampling of individuals is appropriate to determine the subspecies composition via ITS2 sequencing, only 6 specimens were DNA profiled from KV while 37 were profiled from Rabai. What is the reason behind this choice? In order to compare the subspecies composition between the sites, a more balanced design, or at least a larger sample size from KV is necessary. This sample imbalance may also explain why Ae. lilii was only found in Rabai and not in KV.

4. The conclusion that Ae. lilii is of minimal epidemiological importance is an overstatement, since is based on a small sample size and one collected specimen that was not blood-fed. Sample size and trap type could have influenced these results. This point should be discussed.

5. The authors mention in passing the possibility of a new cryptic species, but they do not elaborate or indicate which sample are they referring to. It is difficult to see from Figure 2, but maybe they are referring to samples KV_BF7,4 and 6?

Minor comments:

Figure 1: include a small insert of a map of Africa with the location of Kenya.

Figure 1 legend mentions “Kajulu”, but the location of Kajulu is not indicated in the image.

Figure 3: present the results by members of the complex, or only present Ae. bromeliae.

Figure 4: combine with Figure 2 and indicate the number of blood feed mosquitoes used in each of the bar graphs to the right.

Line 150 and elsewhere: the use of molecular “speciation” is incorrect. Speciation refers to the evolution of a new species. In this case, the authors are referring to a method for species identification using molecular data. I suggest to use “molecular identification” or “species typing”.

Line 180: eliminate “however”
