# Supplementary material for: Survival rate, blood feeding habits and sibling species composition of Aedes simpsoni complex: Implications for arbovirus transmission risk in East Africa
Source: PLoS Negl Trop Dis. 2022 Jan 24;16(1):e0010171. doi: 10.1371/journal.pntd.0010171 (PMC8812930; doi:10.1371/journal.pntd.0010171)
Supplement: Attachment — Submitted filename: Comments on Influence of Vectoral capacity and genetic structure .docx [file pntd.0010171.s002.docx]

**General comments**

This study reports survey carried out in some villages in Kenya to understand the vectorial capacity and genetic structure of mosquito members of *Aedes simpsoni* group, a group comprising highly important, albeit neglected, YF vectors such as *Ae. bromeliae*. Although the data reported are relevant to broaden our undertesting of YF and associated arbovirus transmission ecological system, I think there are major aspect that must be addressed before this manuscript is considered for publication either in Plos Neglect or elsewehere.

In my understanding the manuscript title is highly misleading as the study hasn’t actually investigate the influence of vectorial capacity and genetic structure of *Ae. simpsoni* on arbovirus transmission. What author did was applying molecular analysis tools to investigate species composition of *Ae. simpsoni* complex. By reading the title I was expecting to find in the main manuscript description information about the amount and distribution of genetic variability between members of the *Ae. simpsoni* complex and possible link with their ability or not of transmitting arbovirus (YF) at study site. Additionally, the data reported doesn’t allow authors to make any inference about vectorial capacity as they only investigated mosquito blood-feeding habits and survival rate. Therefore, further information needs to be gathered so authors can really estimate vectorial capacity, these information include human-biting density (***m***), which cannot be inferred by a CO2-baited BG-traps, and human-biting habits (***a***). The product of these two parameters is the human-biting rate (**ma**). The human biting habit (***a***) is a product of human biting frequency (i.e., the length of the gonotrophical cycle) and the human blood index. As such, the manuscript doesn’t show convincingly that *Ae. bromeliae* has higher vectorial capacity although author have concluded that way. The inference of vectorial capacity has been done based solely on estimates of mosquito survival rate and human blood-feeding habits, lacking other fundamental parameters such as those aforementioned. Furthermore, no investigation of arbovirus infection was conducted on collected samples.

A such, in my perception, this study reports basically the occurrence, species composition, survival rate and blood-feeding habitats of *Ae. simpsoni* complex members. Therefore, result should be reported and discussed considering only the data produced and possible limitations for making generalization given the type of sampling approach applied and relatively lower number of specimens analysed (N= 539). For instance, only 27 blood-fed mosquitoes were analysed to determine the source of blood-meal. No serious conclusion can be made with such a smaller sample size.

**Abstract**

Abstract lines 34-36, “We here, address the need for improved understanding of vectorial capacity and genetic influence of *Ae. simpsoni* mosquitoes. Do the authors mean genetic influence of genetic structure?

Line 37- “Age structure was first estimated by parity in *Ae. simpsoni*”. This should be rewritten to read Age structure was determined based on the appearance of mosquito ovaries observed by dissection.

Lines 37-38 not clear for what purpose PCR and sequencing was used. What gene(s) or genetic markers were targeted?

Lines 39-41, Inference on vectorial capacity is not that simple, High human blood index doesn’t necessarily mean the species in question has consequently high vectorial capacity. Vectorial capacity is also a direct function of mosquito density relative to humans and human-biting habits (obtained by the product of frequency of mosquito biting and the human blood index also known as index of anthropophagy. This study doesn’t estimate any realistic vectorial capacity, it investigated blood-feeding habits and survival rates of *Ae. simpsoni* complex sibling species.

**Introduction**

This paper is about the influence of vectorial capacity and genetic structure of *Aedes simpsoni* on arbovirus transmission risk. However, I cannot see the rationale of studying the influence of vectorial capacity and genetic structure of *Ae. simpsoni* on transmission of arbovirus clearly presented in the introduction, particularly the extent to which arbovirus transmission can be influenced by vectorial capacity and genetic structure. The introduction emphasizes more aspects related to *Ae. simpsoni* bionomics, and virtually none account about the role of genetic structure on arbovirus transmission has been menioned. Moreover, it is not clearly stated what aspects of genetic structure authors have investigated. In fact, the influence of genetic structure hasn’t been mentioned has one of this study goals. It is clear to me that the goal was to study *Ae. simpsoni* survival rate and blood-feeding pattern, as it has been stated in lines 92-93.

*Aedes simpsoni* has been referred interchangeably as *Ae. simpsoni s.l.* and *Ae. simpsoni*. *Ae. simpsoni* has been sometimes referred to as *Ae. simpsoni s.s* which makes the reading difficulty to follow. Therefore, effort should be done to write the species name clearly so readers can understand the authors points.

Lines 77 – 78, “…last Kenyan YF outbreak”. I would suggest replacing the words “last Kenyan YF outbreaks” by one of the major YFV outbreak observed in Kenya so far (1992 - 95), as it looks to me that those were the final YF outbreak in Kenya, no outbreak will happen again in the future. This kind of contradicts what has been written in a previous paragraph (lines 72-73), concerning importation of YF cases from RDC and Angola into Kenya, in 2015-2016.

Line 80- “and this leaves the fate of East Africa unknown”. Suggest replacing by whether the East Africa region may face same fate in the future remains uncertain

Line 85- I think the correct spelling is *Ae. lilii*

**Material and Methods**

Local climate and environmental/landscape features of studied sites that may promote *Ae. simpsoni s.l* occurrence and possibly influence mosquito bionomics should be described, such as temperature, rainfalls pattern, humidity, etc, apart from tree holes and plat axile. Since this study has to do with vectorial capacity, which in turns depends on mosquito blood habits and host availability, a general account of human and other domestic animals census data should be described too.

**Mosquito survey**

Adult host-seeking female mosquitoes were surveyed using CO2-baited BG Sentinel traps at three trapping periods after the rains (lines 124-125). Clarify what do these trapping periods consisted about and why samplings were conducted only after rains? No details have been provided to clarify the procedure used to assign the traps to different sites.

Lines 126-129, inform of daily variations of temperature, rainfalls, humidity are results actually, so they should be placed in a specific section of the results.

Line 130 - Traps were set during the day (06:30 – 18:00h). Mosquito sampling as performed from morning to evening (06:30 – 18:00).

**Parity rate, daily survival and longevity estimation**

It is not clear whether mosquitoes were dissected after preservation or before preservation at -80ºC.

**Statistical analyses**

Proper statistical analysis to determine the degree of variability of genetic structure between species should be conducted as well.

**Results**

**Parity, daily survival and age estimates**

I will ask authors to refrain from making statistical analysis to determine the significance of difference between data from different locations when the sampling timings are not similar because it is illogical. For instance, in Rabai the data used to estimate parity rate are from August-September 2019 and February 2020, whereas in KV there was only data from November 2019. There is no point here doing any sort of comparison to determine difference between sites. Do only descriptive analysis that should be restricted to each site. Don’t do any site-to-site comparison because the data were obtained in different contexts, there is no room for spatial comparisons with these data.

**Genetic composition of parous *Ae. simpsoni s.l*.**

This study didn’t characterize mosquito genome, what you did was conducting molecular identification of *Ae. simpsoni* sibling species by analysing the ITS2 region gene sequences. So, for clarity consider changing the title so it can read

**Species composition of *Ae. simpsoni* complex**.

Line 215, specify a subset of what sample size? How were the sample subsets selected?

Line 218 “…suggesting predominance of this species in both areas”. This is a discussion, not a result. Place it somewhere in the discussion.

**Blood meal feeding patterns**

Line 231 – indicate the total blood-fed mosquitoes from KV and Rabai that together made up 27 blood-fed specimens.

**Discussion**

The authors discuss the implications of their findings regarding arbovirus transmission risk at study sites, arguably due to high abundance of *Ae. bromeliae*, one of the most relevant YF vector in East African region. However, the authors have failed to emphasize while discussing result-by-result that in general the overall size of the mosquito sample was too small, that is, only 539 specimens were collected. This obviously precludes from making any strong conclusion regarding the variables analysed, such as, parity rate and longevity and source of blood meal. No strong inference can be also done on feeding habit when only 27 mosquitoes were analysed. The same applies to survival rate and longevity which also require observation of large number of dissected ovaries. Therefore, the results should be described and discussed very cautiously.

Lines 290 – 291 “The very low numbers and absence of blood feeding for *Ae. lilii* found in the current study confirms previous reports for the prevalence of this subspecies in eastern Africa”. There is no clear connection between this paragraph and the one before it. Consider rewriting or deleting.

Lines 294- 296, the authors implies that more sibling species may exist in the *Ae. simpsoni* group apart from those widely known three species. Please elaborate?

**Conclusion**

This section reflects the data reported
