# Supplementary material for: Survival rate, blood feeding habits and sibling species composition of Aedes simpsoni complex: Implications for arbovirus transmission risk in East Africa
Source: PLoS Negl Trop Dis. 2022 Jan 24;16(1):e0010171. doi: 10.1371/journal.pntd.0010171 (PMC8812930; doi:10.1371/journal.pntd.0010171)
Supplement: Attachment — Submitted filename: Review comments Jan522.docx [file pntd.0010171.s003.docx]

5^th^ January 2022

Editor-in-Chief,

PLoS Neglected Tropical Diseases,

Shaden Kamhawi (PhD).

Dear Prof Kamhawi,

**Re: Manuscript re-submission:** PNTD-D-21-01450

We are pleased to resubmit the manuscript entitled **" Influence of vectoral capacity and genetic structure of *Aedes simpsoni* on arbovirus transmission risk in Ea****st Africa**" for further consideration for publication in your journal. We have made all the changes you suggested in your letter and addressed the comments of the reviewers in the notes below. Please note that our responses are indicated in type italics below the reviewer’s comment.

Yours sincerely,

David P. Tchouassi

Corresponding author, email: [dtchouassi@icipe.org](mailto:dtchouassi@icipe.org)

Dear Dr. Tchouassi & co-authors,

Many thanks for submitting your manuscript to PLOS NTD. Apologies for the delay in getting this manuscript reviewed. Your manuscript has now been evaluated by three reviewers. The Reviewers agree that the study is of interest and all three provide feedback to improve the manuscript. I would like to ask you to respond to the concerns raised by the Reviewers and revise the manuscript accordingly (major revisions requested). All three reviewers pointed out that the manuscript in its current form, cannot draw firm conclusions regarding vectorial capacity since e.g. the vector competence for YFV of the mosquito species in question is unknown. It would be wonderful if the authors could provide experimental data to assess vector competence. However, if such data cannot be provided in this revision, the title and text of the manuscript should be modify to reflect this.

Many thanks for your hard work - I look forward to seeing a revised version of this manuscript.

all the best,

Felix


Reviewer's Responses to Questions

<b>Key Review Criteria Required for Acceptance?</b></br></br>
As you describe the new analyses required for acceptance, please consider the following:</br></br>

<b>Methods</b></br></br>
-Are the objectives of the study clearly articulated with a clear testable hypothesis stated?</br>
-Is the study design appropriate to address the stated objectives?</br>
-Is the population clearly described and appropriate for the hypothesis being tested?</br>
-Is the sample size sufficient to ensure adequate power to address the hypothesis being tested?</br>
-Were correct statistical analysis used to support conclusions?</br>
-Are there concerns about ethical or regulatory requirements being met?</br></br>

**Reviewer #1: Objectives are clearly articulated and methods used are appropriate. Sample size is small and imbalanced for certain analysis, and thus prevents some statistical comparisons. However, the data is still informative.**

**Reviewer #2: The methods are adequate and well described. Sample size and statistics are correct.
However, the study design does not completely test the hypothesis of vector capacity. vector capacity encompasses vector competence and mosquito behavior and the study does not contain a measure of vector competence. Furthermore, there is no mention of earlier evaluation of the Ae bromeliae vector competence.**

*Re:* *We totally agree with the concerns raised regarding the subject of vectorial capacity (VC) which can be quite complex and includes aspects of vector ecology, genetics and behavior factors. In fact, the original concept of VC as defined by Garett-Jones et al stresses parameters such as abundance (m), host feeding (a), vector longevity (p), and EIP (n) and did not include vector competence. This original equation has sometimes been revised by different authors and in some cases to include vector competence. This is especially important in arbovirus transmission context which must involve competent vectors. Undeniably, human blood feeding and survivorship (which our present study focused on based on existing knowledge gaps) are the two most sensitive parameters of VC with vector competence being of relatively minor importance (Reviewed by Lounibos and Kramer, JID 2016;214(S5):S453–8). The competence of Ae. bromeliae is inferred from literature widely known as the main yellow fever vector although this was not verified/confirmed in the present study. The current study did not investigate vector competence of Ae. bromeliae. We have, nonetheless, included results from the only known evaluation of Ae. simpsoni s.l.* vector competence to YFV in our discussion. L279-280.

**Reviewer #3: Local climate and environmental/landscape features of studied sites that may promote Ae. simpsoni s.l occurrence and possibly influence mosquito bionomics should be described, such as temperature, rainfalls pattern, humidity, etc, apart from tree holes and plant axils. Since this study has to do with vectorial capacity, which in** **turns depends on mosquito blood habits and host availability, a general account of human and other domestic animals census data should be described too.**

*Re:* *Data relating to temperature, rainfall and humidity during the trapping periods were provided (L..). We have included the following in the site description as well:* *Kerio Valley is an arid to semi-arid ecology within the Rift Valley that is sparsely populated. The main economic activities of the inhabitants include farming of crops like maize and cotton, and livestock keeping especially goats and cattle. Rabai is one of the seven administrative sub-counties of Kilifi County in the coast of Kenya. The main economic activities in the area include subsistence agriculture, casual labor, crafts and petty trading. An account of the human density in these areas was given in L107-108. Information on main activities in the areas including livestock keeping has been included, although actual data on the density of domestic animal is currently unavailable (L114-118).*

**Mosquito survey**
**Adult host-seeking female mosquitoes were surveyed using CO_2_-baited BG Sentinel traps at three trapping periods after the rains (lines 124-125). Clarify what do these trapping periods consisted about and why samplings were conducted only after rains?** **No details have been provided to clarify the procedure used to assign the traps to different sites.**

*Re:* *Sampling was conducted after the rains to ascertain abundance of mosquitoes important for arbovirus circulation. This sentence has now been rephrased to read “Adult host-seeking female mosquitoes were surveyed using CO_2_-baited BG Sentinel traps at three trapping periods. In each area, the trapping exercise began about three weeks following continuous rainfall for at least a week. This timing was critical to maximize the assessment of abundance considering the predominant tree hole and plant axils breeding ecology of Ae. simpsoni s.l.” (L130-133). More information has been added on the procedure used to assign the traps to the different site. Sampling was designed to cover a large spatial area within each site. Traps were placed outdoors in the vegetation around human habitations and moved every other day to a new locality at least 500 m away (L140-142).*

**Lines 126-129, inform of daily variations of temperature, rainfalls, humidity are results actually, so they should be placed in a specific section of the results.**

*Re: We believe this information describes better the local weather patterns during sampling at the study sites and preferably to leave here.*

**Line 130 - Traps were set during the day (06:30 – 18:00h). Mosquito sampling as performed from morning to evening (06:30 – 18:00).**

*Re: This has been revised and now reads:* *Mosquito sampling was conducted from morning to evening (06:30 – 18:00) on the same day. L138*

**Parity rate, daily survival and longevity estimation
It is not clear whether mosquitoes were dissected after preservation or before** **preservation at -80ºC.**

*Re: Dissection was performed after preservation. L148*

**Statistical analyses
Proper statistical analysis to determine the degree of variability of genetic structure between species should be conducted as well.**

*Re: This is not within the scope of the present study which was limited to discriminating the probable sibling species in Ae. simpsoni s.l. by pcr of the ITS2 region, followed by sequencing and phylogenetic reconstructions.*

**<b>Results</b></br></br>
-Does the analysis presented match the analysis plan?</br>
-Are the results clearly and completely presented?</br>
-Are the figures (Tables, Images) of sufficient quality for clarity?</br></br>

Reviewer #1: (No Response)

Reviewer #2: The results are clearly and completely presented. However, as mentioned for the methods, the study would greatly benefit from a measure of vector competence (by oral feeding Ae bromeliae with YFV) to meet their objective and main message of identifying Ae bromeliae as a capable vector for YFV.**

*Re: Please, refer to our response to the query above. To strengthen our conclusions, we have also referenced the only known Ae. simpsoni s.l vector competence study. L279*

**Reviewer #3: Parity, daily survival and age estimates
I will ask authors to refrain from making statistical analysis to determine the significance of difference between data from different locations when the sampling timings are not similar because it is illogical. For instance, in Rabai the data used to estimate parity rate are from August-September 2019 and February 2020, whereas in KV there was only data from November 2019. There is no point here doing any sort of comparison to determine difference between sites. Do only descriptive analysis that should be restricted to each site. Don’t do any site-to-site comparison because the data were obtained in different contexts, there is no room for spatial comparisons with these data.**

*Re: Besides, descriptive statistics, we feel is ok as is comparing findings of parity across the different sampling periods. In fact, the period is an important predictor of interest given that the trapping designs were similarly applied across sites.*

**Genetic composition of parous Ae. simpsoni s.l.
This study didn’t characterize mosquito genome, what you did was conducting molecular identification of Ae. simpsoni sibling species by analysing the ITS2 region gene sequences. So, for clarity consider changing the title so it can read Species composition of Ae. simpsoni complex.**

*Re: Without genetic analysis of the samples, the true composition of the sibling species will not be known, and this section reflects that. The title of the manuscript has now been revised to include the suggestion of the reviewer.*

**Line 215, specify a subset of what sample size? How were the sample subsets selected?**

*Re: The samples were randomly selected (logistical constraints relating to costs involved in sequencing contributed to the analysis of this number)*

**Line 218 “…suggesting predominance of this species in both areas”. This is a discussion, not a result. Place it somewhere in the discussion.**

*Re: deleted here as suggested.*

**Blood meal feeding patterns**
**Line 231 – indicate the total blood-fed mosquitoes from KV and Rabai that together made up 27 blood-fed specimens.**

*Re: Information now captured. L242, 243*

**Discussion
The authors discuss the implications of their findings regarding arbovirus transmission risk at study sites, arguably due to high abundance of Ae. bromeliae, one of the most relevant YF vector in East African region. However, the authors have failed to emphasize while discussing result-by-result that in general the overall size of the mosquito sample was too small, that is, only 539 specimens were collected. This obviously precludes from making any strong conclusion regarding the variables analysed, such as, parity rate and longevity and source of blood meal. No strong inference can be also done on feeding habit when only 27 mosquitoes were analysed. The same applies to survival rate and longevity which also require observation of large number of dissected ovaries. Therefore, the results should be described and discussed very cautiously.**

***Re:*** *Mention is made of the need for large samples required for estimation of parity. By using archived samples, we had little control on the sample size. Nonetheless, the sample size compares or even exceeds those used for estimating survival by parity for Aedes species and other mosquitoes (See Sang et al., 2010: Am J Trop Med Hyg 83(2 Suppl), 28–37; dos Santos Andrade et al 2019:* *J Vector Ecol. 2019;44(2):233-40; Pan et al., 2012: Parasit Vectors, 5, 136; Moreno et al 2017; PLoS Negl Trop Dis 11(2): e0005337) so, should provide a reliable estimate of survival. Furthermore, the samples may in fact reflect the abundance of the species in the different areas. The species abundance (possibly attributed to the trapping designed employed) is a significant increase compared to that recorded in a previous study where only 29 specimens of this species was encountered from a trapping effort of about 180 traps (Agha et al 2017: Parasites & Vectors, 10:628).*

*We acknowledged the issue regarding sample size used for blood meal analysis in the discussion. It is good to note that previous attempts to target resting mosquito collections on vegetation using prokopack aspirators was largely unsuccessful (Agha et al 2017: Parasites & Vectors, 10:628). This challenge does not preclude the lack of enough sampling effort but reflects the difficulty in sampling enough blood fed mosquitoes of this species. This challenge was highlighted in the discussion section.*

**Lines 290 – 291 “The very low numbers and absence of blood feeding for Ae. lilii found in the current study confirms previous reports for the prevalence of this subspecies in eastern Africa”. There is no clear connection between this paragraph and the one before it. Consider rewriting or deleting.**

*Re: This sentence has been deleted as suggested*

**Lines 294- 296, the authors implies that more sibling species may exist in the Ae. simpsoni group apart from those widely known three species. Please elaborate?**

*Re: This sentence is preceded by one describing phylogenetic analysis illustrating other potential species that did not cluster with the known species in the complex (clades II and III), implying greater diversity.*


**<b>Conclusions</b></br></br>
-Are the conclusions supported by the data presented?</br>
-Are the limitations of analysis clearly described?</br>
-Do the authors discuss how these data can be helpful to advance our understanding of the topic under study?</br>
-Is public health relevance addressed?</br></br>

Reviewer #1: (No Response)

Reviewer #2: The study aims to provide information about the vectors for YFV in east africa. The authors collected mosquitoes from 3 geographically distinct areas in Kenya. The mosquito species was identified, their parity rates and the origin of the blood (human or other animals). These information incriminate Ae bromiliae from the Ae. simpsoni complex as a vector for arboviruses in these areas. However, there is a lack of information about the vector competence for Ae. bromaliae for the major arboviruses. From a quick literature search that was not available in the introduction, I found that Ae bromeliae vector competence is partially known although the available studies date back some time ago.
The current study would be more informative about the potential role of Ae bromeliae if they included vector comptence data. Importantly, the authors cannot state that they studied the influence of vector capacity of Ae simpsoni without including a measure of vector competence for YFV.**

*Re: Please, refer to our response to this query indicated above.*

**Reviewer #3: Conclusion
This section reflects the data reported**

*Re: Thank you.*

**<b>Editorial and Data Presentation Modifications?</b></br><br/>
Use  this section for editorial suggestions as well as relatively minor modifications of existing data that would enhance clarity. If the only modifications needed are minor and/or editorial, you may wish to recommend “Minor Revision” or “Accept”.

Reviewer #1: (No Response)**

**Reviewer #2: There is a mispelling in the title: "influence of vectorIal capacity".**

*Re: Thank you. The title has been revised and now reads:* ***Survival rate, blood feeding habits and sibling species composition of Aedes simpsoni complex: implications for arbovirus transmission risk in East Africa***

**l. 63-65: I do not understand this sentence.**

*Re: We feel the sentence is explicit.*

**l. 279-281: I also do not understand this sentence.**

*Re: We have changed ‘YF epidemiological’ to ‘YF virus transmission’*

**l. 235: there is a parenthesis missing.**

*Re: Parenthesis added*

**l. 116: there is another parenthesis missing.**

*Re: Parenthesis added*

Reviewer #3: (No Response)

--------------------

**<b>Summary and General Comments</b></br></br>
Use  this section to provide overall comments, discuss strengths/weaknesses of the study, novelty, significance, general execution and scholarship. You may also include additional comments for the author, including concerns about dual publication, research ethics, or publication ethics. If requesting major revision, please articulate the new experiments that are needed.

Reviewer #1: (No Response)

Reviewer #2: (No Response)

Reviewer #3: This study reports survey carried out in some villages in Kenya to understand the vectorial capacity and genetic structure of mosquito members of Aedes simpsoni group, a group comprising highly important, albeit neglected, YF vectors such as Ae. bromeliae. Although the data reported are relevant to broaden our undertesting of YF and associated arbovirus transmission ecological system, I think there are major aspect that must be addressed before this manuscript is considered for publication either in Plos Neglect or elsewehere.
 In my understanding the manuscript title is highly misleading as the study hasn’t actually investigate the influence of vectorial capacity and genetic structure of Ae. simpsoni on arbovirus transmission. What author did was applying molecular analysis tools to investigate species composition of Ae. simpsoni complex. By reading the title I was expecting to find in the main manuscript description information about the amount and distribution of genetic variability between members of the Ae. simpsoni complex and possible link with their ability or not of transmitting arbovirus (YF) at study site. Additionally, the data reported doesn’t allow authors to make any inference about vectorial capacity as they only investigated mosquito blood-feeding habits and survival rate. Therefore, further information needs to be gathered so authors can really estimate vectorial capacity, these information include human-biting density (m), which cannot be inferred by a CO2-baited BG-traps, and human-biting habits (a). The product of these two parameters is the human-biting rate (ma). The human biting habit (a) is a product of human biting frequency (i.e., the length of the gonotrophical cycle) and the human blood index. As such, the manuscript doesn’t show convincingly that Ae. bromeliae has higher vectorial capacity although author have concluded that way. The inference of vectorial capacity has been done based solely on estimates of mosquito survival rate and human blood-feeding habits, lacking other fundamental parameters such as those aforementioned. Furthermore, no investigation of arbovirus infection was conducted on collected samples.
A such, in my perception, this study reports basically the** **occurrence, species composition, survival rate and blood-feeding habitats of Ae. simpsoni complex members. Therefore, result should be reported and discussed considering only the data produced and possible limitations for making generalization given the type of sampling approach applied and relatively lower number of specimens analysed (N= 539). For instance, only 27 blood-fed mosquitoes were analysed to determine the source of blood-meal. No serious conclusion can be made with such a smaller sample size.**

*Re:* *Please, refer to our response to this query addressed above. The title has been revised and now reads:* ***Survival rate, blood feeding habits and sibling species composition of Aedes simpsoni complex: implications for arbovirus transmission risk in East Africa***

**Abstract**
**Abstract lines 34-36, “We here, address the need for improved understanding of vectorial capacity and genetic influence of Ae. simpsoni mosquitoes. Do the authors mean genetic influence of genetic structure?**

*Re: This sentence has been deleted and the ensuing sentence revised and now reads:* *In this study conducted in two areas with divergent ecosystems: peri-urban (coastal Rabai) and rural (Rift Valley Kerio Valley) in Kenya, survival rate was determined by parity in Ae. simpsoni s.l. mosquitoes sampled using CO_2_-baited BG Sentinel traps. L34-36*

**Line 37- “Age structure was first estimated by parity in Ae. simpsoni”. This should be rewritten to read Age structure was determined based on the appearance of mosquito ovaries observed by dissection.**

*Re: Parity entails dissection and observing the appearance of the ovaries and this is captured in the methods section.*

**Lines 37-38 not clear for what purpose PCR and sequencing was used. What gene(s) or genetic markers were targeted?**

*Re: The purpose*: *to identify the sibling species in the Ae. simpsoni complex* *has been captured. ITS2 as target marker has also been included. L39*

**Lines 39-41, Inference on vectorial capacity is not that simple, High human blood index doesn’t necessarily mean the species in question has consequently high vectorial capacity. Vectorial capacity is also a direct function of mosquito density relative to humans and human-biting habits (obtained by the product of frequency of mosquito biting and the human blood index also known as index of anthropophagy. This study doesn’t estimate any realistic vectorial capacity, it investigated blood-feeding habits and survival rates of Ae. simpsoni complex sibling species.**

*Re: As explained before, blood feeding and survival are two of the most sensitive indicators of vectorial capacity. It is only logical for such inference to be drawn for Ae. bromeliae given its known role in the transmission of YFV in literature. Our conclusions have been further supported by inclusion of a reference to the vector competence data of Ae. simpsoni s.l to YFV in Kenya. L279.*

**Introduction
This paper is about the influence of vectorial capacity and genetic structure of Aedes simpsoni on arbovirus transmission risk. However, I cannot see the rationale of studying the influence of vectorial capacity and genetic structure of Ae. simpsoni on transmission of arbovirus clearly presented in the introduction, particularly the extent to which arbovirus transmission can be influenced by vectorial capacity and genetic structure. The introduction emphasizes more aspects related to Ae. simpsoni bionomics, and virtually none account about the role of genetic structure on arbovirus transmission has been mentioned. Moreover, it is not clearly stated what aspects of genetic structure authors have investigated. In fact, the influence of genetic structure hasn’t been mentioned has one of this study goals. It is clear to me that the goal was to study Ae. simpsoni survival rate and blood-feeding pattern, as it has been stated in lines 92-93.**

*Re: The title of the manuscript has been revised to capture the content of the introduction* *viz:* ***Survival rate, blood feeding habits and sibling species composition of Aedes simpsoni complex: implications for arbovirus transmission risk in East Africa***

**Aedes simpsoni has been referred interchangeably as Ae. simpsoni s.l. and Ae. simpsoni. Ae. simpsoni has been sometimes referred to as Ae. simpsoni s.s which makes the reading difficulty to follow. Therefore, effort should be done to write the species name clearly so readers can understand the authors points.**

*Re: Ae. simpsoni s.s. is only used once to indicate the specific sibling species (L86). All Ae. simpsoni s.l. and further in the manuscript is referred to as Ae. simpsoni (L105).*

**Lines 77 – 78, “…last Kenyan YF outbreak”. I would suggest replacing the words “last Kenyan YF outbreaks” by one of the major YFV outbreak observed in Kenya so far (1992 - 95), as it looks to me that those were the final YF outbreak in Kenya, no outbreak will happen again in the future. This kind of contradicts what has been written in a previous paragraph (lines 72-73), concerning importation of YF cases from RDC and Angola into Kenya, in 2015-2016.**

*Re: Revised and now reads...last documented YF outbreak in Kenya. L77-78*

**Line 80- “and this leaves the fate of East Africa unknown”. Suggest replacing by** **whether the East Africa region may face same fate in the future remains uncertain**

*Re: Thank you. Correction effected as suggested. L..*

**Line 85- I think the correct spelling is Ae. lilii**

*Re: Correction effected. Thank you.*

**PLOS authors have the option to publish the peer review history of their article (<a href="**[**https://journals.plos.org/plosntds/s/editorial-and-peer-review-process#loc-peer-review-history**](https://journals.plos.org/plosntds/s/editorial-and-peer-review-process#loc-peer-review-history)**" target="_blank">what does this mean?</a>). If published, this will include your full peer review and any attached files.<br><br>

If you choose “no”, your identity will remain anonymous but your review may still be made public.<br><br>

<b>Do you want your identity to be public for this peer review?</b> For information about this choice, including consent withdrawal, please see our <a href="**[**https://www.plos.org/privacy-policy**](https://www.plos.org/privacy-policy)**" target="_blank">Privacy Policy</a>.

Reviewer #1: No
Reviewer #2: No
Reviewer #3: No**

**Reviewer 1 additions**

**Summary:**

**The goal of this paper is to evaluate the vectorial capacity of members of the Ae. simpsoni complex in two areas in Kenya: coastal Rabai and Rift Valley Kerio Valley), based on longevity and blood feeding habits. The authors analyze 539 mosquitos collected using CO2-baited BG-sentinel traps between 2019 and 2020, determine their longevity based on parity and use a fragment of the ITS2 to identify a subset of the samples (N=43) to subspecies. The authors also identify the blood-host of 24 blood-fed mosquitoes.**

**The data suggests high survival rates (longevity) for Ae. simpsoni in Kenya, and highlight that Ae. bromeliae is the dominant species in the region. This paper also reports the presence of Ae. lilii for first time in Rabai, Kenya. Blood-meal analysis indicates that members of this complex (mostly Ae. bromeliae) have a wide host feeding range, with moderate feeding from humans. The authors conclude based on longevity and moderate feeding on humans, that Ae. bromeliae has a high vectorial capacity for pathogen transmission. They also mention the possibility of having discovered a new cryptic species, but evidence for this is unclear.**

**Using basic molecular and morphological data, this manuscript provides important information on the ecology of the Ae. simpsoni complex in two sites of Kenya, which is relevant to evaluate vectorial capacity of these mosquitoes as vectors of disease. I find the results interesting and worth publishing; however, I have the concerns listed below.**

**Mayor comments:**

**1. Large numbers of mosquitoes were analyzed for parity and survival rate, yet mean longevity rate is reported without confidence intervals. It will be interesting to know the distribution of this parameter. Also, is it possible that the high survival rates derived from parity are consequence of a bias in the trapping method? Furthermore, the assumption of a 3-day gonotrophic cycle can significantly impact this metric, how realistic is this value? Can the authors estimate the length of the gonotrophic cycle under laboratory conditions? The citation provided in the text is for the gonotrophic cycle of a different mosquito species.**

*Re: We have estimated confidence intervals (CI) around parous rates for the different sampling periods and now included in the text (L…). Unfortunately, literature on gonotrophic cycle is not available for the target mosquito. The difficulty in colonizing this mosquito species could explain this. We have clearly justified that the survival estimate is based on an assumed value for the gonotrophic cycle(n=3) inferred from another Aedes mosquito.*

**2. Out of the 539 mosquitoes screened for parity, a subset of 43 were used for species identification by ITS2 and used to construct the ML tree from figure 2. Then, another ML tree from ITS2 sequences is presented in Figure 4, this time including blood-fed specimens. It is not clear to me if the 24 blood-fed mosquitoes were included among the 43 individuals from Figure 2. I do not see a benefit on having two independent figures providing basically the same information. I suggest to combine all sequences from Figure 2 and 4 into one figure, with one ML tree and including the information of the host. Also, it is very difficult to see which samples belong to which clade from Figure 2, expanding the tree along the horizontal axis may help.**

*Re: Thank you for the concern. Simpler is better and we feel is ok to leave the figures as they are. Fig 2 has been expanded along the horizontal axis as suggested to improve clarity.*

**3. Unbalanced sampling. While subsampling of individuals is appropriate to determine the subspecies composition via ITS2 sequencing, only 6 specimens were DNA profiled from KV while 37 were profiled from Rabai. What is the reason behind this choice? In order to compare the subspecies composition between the sites, a more balanced design, or at least a larger sample size from KV is necessary. This sample imbalance may also explain why Ae. lilii was only found in Rabai and not in KV.**

*Re: This is a valid concern and a weakness in sample selection. However, we have been careful not to make comparisons on the extent of species diversity between Rabai and KV and acknowledged the need for further studies (L295).*

**4. The conclusion that Ae. lilii is of minimal epidemiological importance is an overstatement, since is based on a small sample size and one collected specimen that was not blood-fed. Sample size and trap type could have influenced these results. This point should be discussed.**

*Re: This is true. We provided only a suggestion based on the available data.*

**5. The authors mention in passing the possibility of a new cryptic species, but they do not elaborate or indicate which sample are they referring to. It is difficult to see from Figure 2, but maybe they are referring to samples KV_BF7,4 and 6?**

*Re: This has been clarified as clades II and III (Fig 4)..L304*

**Minor comments:**

**Figure 1: include a small insert of a map of Africa with the location of Kenya.**

*Re: Map revised as suggested*

**Figure 1 legend mentions “Kajulu”, but the location of Kajulu is not indicated in the image.**

*Re: The counties for the sampling sites are indicated on the map only; these sites are captured in the legend.*

**Figure 3: present the results by members of the complex, or only present Ae. bromeliae.**

*Re: Fig 4 is a breakdown of Fig 3 after analysis to define the sibling species.*

**Figure 4: combine with Figure 2 and indicate the number of blood feed mosquitoes used in each of the bar graphs to the right.**

*Re: Please, refer to our response to this query provided above*

**Line 150 and elsewhere: the use of molecular “speciation” is incorrect. Speciation refers to the evolution of a new species. In this case, the authors are referring to a method for species identification using molecular data. I suggest to use “molecular identification” or “species typing”.**

*Re: Thank you. Correction effected.*

**Line 180: eliminate “however”**

*Re: Deleted*
