# Supplementary material for: Survival rate, blood feeding habits and sibling species composition of Aedes simpsoni complex: Implications for arbovirus transmission risk in East Africa
Source: PLoS Negl Trop Dis. 2022 Jan 24;16(1):e0010171. doi: 10.1371/journal.pntd.0010171 (PMC8812930; doi:10.1371/journal.pntd.0010171)
Supplement: Attachment — Submitted filename: Response to reviewer comments Jan1322.docx [file pntd.0010171.s004.docx]

13^th^ January 2022

Editor-in-Chief,

PLoS Neglected Tropical Diseases,

Shaden Kamhawi (PhD).

Dear Prof Kamhawi,

**Re: Manuscript re-submission:** PNTD-D-21-01450

We are pleased to resubmit the manuscript entitled **" Survival rate, blood feeding habits and sibling species composition of *Aedes simpsoni complex*: implications for arbovirus transmission risk in East Africa**" for further consideration for publication in your journal. We have made all the changes you suggested in your letter and addressed the comments of the reviewers in the notes below. Please note that our responses are indicated in type italics below the reviewer’s comment.

Yours sincerely,

David P. Tchouassi

Corresponding author, email: [dtchouassi@icipe.org](mailto:dtchouassi@icipe.org)

**Dear authors,

Many thanks for revising the manuscript and answering the Reviewer's questions. I have some very minor comments that need to be addressed before acceptance, please find them below:

PNTD-D-21-01450_R1

Feedback on revised MS**

**Abstract:
lines 42-43 “and consequently, high vectorial capacity for pathogen transmission”: please rephrase ‘consequently’ to ‘potentially’ or similar. As the Reviewers indicated, caution should be taken in this study with regards to conclusions related to vectorial capacity (especially in the abstract). The word ‘consequently’ should therefore be revised. Same for line 72 in Author Summary.**

*Re: Thank you. This has been effected accordingly. L41, 61*

**MM
line 144, missing space between with and trapping.**

*Re: Thank you. Correction effected. L130.*

**Results:
line 279, parenthesis missing after mongoose.**

*Re: Thank you. Correction effected. L246.*
